# A 4-Gene Signature Associated With Recurrence in Low- and Intermediate-Risk Endometrial Cancer

**DOI:** 10.3389/fonc.2021.729219

**Published:** 2021-08-17

**Authors:** Diocésio Alves Pinto de Andrade, Luciane Sussuchi da Silva, Ana Carolina Laus, Marcos Alves de Lima, Gustavo Nóriz Berardinelli, Vinicius Duval da Silva, Graziela de Macedo Matsushita, Murilo Bonatelli, Aline Larissa Virginio da Silva, Adriane Feijó Evangelista, Jesus Paula Carvalho, Rui Manuel Reis, Ricardo dos Reis

**Affiliations:** ^1^InORP ONCOCLÍNICAS Group, Oncology Institute of Ribeirão Preto, Ribeirão Preto, Brazil; ^2^Molecular Oncology Research Center, Barretos Cancer Hospital, Barretos, Brazil; ^3^Epidemiology and Biostatistics Nucleus, Barretos Cancer Hospital, Barretos, Brazil; ^4^Departament of Pathology, Barretos Cancer Hospital, Barretos, Brazil; ^5^Discipline of Gynecology, Instituto do Cancer do Estado de São Paulo (ICESP), Faculdade de Medicina da Universidade de São Paulo, São Paulo, Brazil; ^6^Life and Health Sciences Research Institute (ICVS), School of Medicine, University of Minho, Braga, Portugal; ^7^ICVS/3B’s - PT Government Associate Laboratory, Braga/Guimarães, Portugal; ^8^Department of Gynecologic Oncology, Barretos Cancer Hospital, Barretos, Brazil

**Keywords:** low- and intermediate-risk endometrioid endometrial carcinoma, genetic signature, recurrence risk score, biomarkers, Brazil

## Abstract

**Background:**

The molecular profile of endometrial cancer has become an important tool in determining patient prognosis and their optimal adjuvant treatment. In addition to The Cancer Genome Atlas (TCGA), simpler tools have been developed, such as the Proactive Molecular Risk Classifier for Endometrial Cancer (ProMisE). We attempted to determine a genetic signature to build a recurrence risk score in patients diagnosed with low- and intermediate-risk endometrial cancer.

**Methods:**

A case-control study was conducted. The eligible patients were women diagnosed with recurrence low- and intermediate-risk endometrial cancer between January 2009 and December 2014 at a single institution; the recurrence patients were matched to two nonrecurrence patients with the same diagnosis by age and surgical staging. Following RNA isolation of 51 cases, 17 recurrence and 34 nonrecurrence patients, the expression profile was determined using the *nCounter^®^ PanCancer Pathways Panel*, which contains 770 genes.

**Results:**

The expression profile was successfully characterized in 49/51 (96.1%) cases. We identified 12 genes differentially expressed between the recurrence and nonrecurrence groups. The ROC curve for each gene was generated, and all had AUCs higher than 0.7. After backward stepwise logistic regression, four genes were highlighted: *FN1, DUSP4, LEF1, and SMAD9*. The recurrence risk score was calculated, leading to a ROC curve of the 4-gene model with an AUC of 0.93, sensitivity of 100%, and specificity of 72.7%.

**Conclusion:**

We identified a four-gene signature that may be associated with recurrence in patients with low- and intermediate-risk endometrial cancer. This finding suggests a new prognostic factor in this poorly explored group of patients with endometrial cancer.

## Introduction

Endometrial cancer is the most prevalent gynecological tumor in developed countries, such as the USA and members of the European Union (1). The number of cases in the last decade have increased, possibly due to the increase in obesity in these countries (2). In Brazil, endometrial cancer is the eighth most commonly diagnosed cancer in women, with 6,540 new cases in 2020 (3). When diagnosed at an early stage, these patients have an excellent prognosis. Countless risk stratifications associate staging with other variables, such as tumor grade, lymphovascular space invasion (LVSI) and histology, to define sequential adjuvant treatments (4).

Traditionally, it has been considered two distinct diseases since Bokhman’s publication in the early 1980s (low-grade endometrioid adenocarcinomas (type I, “well-differentiated”) and nonendometrioid carcinomas (type II, “poorly differentiated”) (5). Recently, The Cancer Genome Atlas (TCGA) project changed the understanding of the carcinogenesis of this tumor, leading to four molecular subgroups with different prognoses (DNA polymerase epsilon (*POLE*) ultramutated, microsatellite instability (MSI) hypermutated, copy number (CN) low, and CN high) (6). Due to the complexity (whole genome sequencing, exome sequencing, microsatellite assays, and CN aberration analysis), costs, and need for ideally frozen tissue for reproducibility of this classification in clinical practice, new methodologies have been developed. The two most currently used are the Proactive Molecular Risk Classifier for Endometrial Cancer (ProMisE) and Leiden/TransPORTEC classification, in which the four groups with different prognoses are also described (7, 8). Immunohistochemistry was used to detect the presence/absence of mismatch repair (MMR) proteins and to evaluate *TP53* mutations, and only one step used genetic sequencing (next-generation or Sanger sequencing) to identify *POLE* hotspot exonuclease domain mutations (7, 8).

Using the TCGA consortium database, some studies have built prognostic models of endometrial cancer recurrence according to genetic signatures or evaluated RNA expression (9, 10). Furthermore, other studies correlate potential genetic signatures with histopathological markers such as tumor-infiltrating immune cells (11, 12).

The aim of this study was to determine a genetic signature of recurrence risk in patients diagnosed with low- and intermediate-risk endometrial cancer in routine formalin-fixed paraffin-embedded (FFPE) tissue using a large panel of 770 genes covering 13 key cancer-related pathways by NanoString, a highly sensitive and robust methodology for RNA expression of FFPE samples.

## Material and Methods

### Patients and Specimens

From a retrospective cohort of 195 patients diagnosed with low- and intermediate-risk endometrial cancer between January 2009 and December 2014 at Barretos Cancer Hospital (BCH), two pathologists with oncogynecologic expertise reviewed the initial report of all patients to confirm their diagnosis. Clinical and pathological features were obtained from the medical records. Of the 22 patients who initially presented recurrence, the diagnosis remained low- and intermediate-risk endometrial cancer for 17 patients. We define low- and intermediate-risk endometrial cancer according to the European Society of Medical Oncology (ESMO)-modified criteria (4). Based on these results, a case-control study was carried out. Nonrecurrence patients with the same histopathological diagnosis were matched to recurrence cases in a 2:1 ratio by age and FIGO (International Federation of Gyneocologic and Obstetrics) staging (IA and IB). Overall, 51 patients (17 recurrence and 34 non-recurrence) were analyzed.

This study was conducted following the ethical principles of the Declaration of Helsinki, and the BCH Ethical Review Board approved it in March 2017 (Reference number 1.942.488).

### DNA and RNA Isolation

DNA and RNA were isolated from 10 µm-thick formalin-fixed paraffin-embedded (FFPE) tumor samples sectioned on slides, as previously reported (13). One slide was stained with hematoxylin and eosin (H&E) and evaluated by experienced pathologists for identification, sample adequacy assessment, and selection of the tumor tissue area (minimum of 60% tumor area). DNA and RNA were isolated using the QiaAmp DNA Micro kit (Qiagen, Hilden, Germany) and the RecoverAll™ Total Nucleic Acid Isolation kit (Ambion by Life Technologies, Austin, TX, USA), respectively. The quality and concentration of DNA and RNA were measured by both a NanoDrop ND-200 spectrophotometer (NanoDrop Products, Wilmington, DE, USA) and Qubit Fluorometric Quantitation (Thermo Fisher Scientific, USA). The samples were stored at -80°C until molecular analysis.

### ProMisE Evaluation

The ProMisE assessment was performed using molecular methodologies, namely, molecular evaluation of MSI, *TP53* mutation analyses by next-generation sequencing, and *POLE* hotspot mutations by Sanger sequencing.

To define MSI, we performed hexaplex PCR with six monomorphic mononucleotide markers (NR21, NR24, NR27, BAT25, BAT26, and HSP110), followed by fragment analysis in a 3500XL Genetic Analyzer sequencer (Applied Biosystems, Foster City, CA, USA), as previously described by our group (14). The presence of two or more markers with instability classified the cases as high microsatellite instability (MSI-H), the presence of one marker with instability was classified as low MSI (MSI-L), and the absence of any marker with instability as microsatellite stable (MSS). Presence of MSI was determined only for MSI-H cases.

To evaluate *POLE* mutations, we used direct Sanger sequencing, as described by Britton et al. PCR was performed using targeted primers for the exonuclease domain (exons 9-14) of *POLE* (15). The purified samples were subjected to capillary electrophoresis in a 3500XL Genetic Analyzer sequencer (Applied Biosystems, Foster City, CA, USA), and the results were analyzed with SegScape v2.7 software (Applied Biosystems, Foster City, CA, USA).

*TP53* mutations were detected with an NGS-based assay using the Illumina TruSight Tumor 15 (TST15) on the MiSeq instrument (Illumina, San Diego, CA, USA) according to the manufacturer’s instructions, as previously reported (16). The TST15 panel assesses all coding sequences of the *TP53* gene. Read alignment and variant calling were performed with BaseSpace BWA Enrichment version 2.1 (Illumina, San Diego, CA, USA) and Sophia DDM^®^ software version 5.7.3 (Sophia Genetics SA, Switzerland). The identification of pathogenic variants occurred after the application of filters to remove low-quality variants. Variants with < 500X read depth, VAF <10%, and intronic, intergenic, 3’ UTR, and synonymous variants were excluded. Thereafter, the variants that presented as polymorphisms, within a frequency >1% in the GnomAD database, were removed (those that had no population frequency information followed in the analyses). Finally, the pathogenicity of variants was checked in the databases ClinVar, IARC TP53, COSMIC, and CGI.

### NanoString nCounter Analysis

Samples were processed for analysis on the NanoString nCounter Flex system using the 770 gene nCounter^®^ PanCancer Pathways Panel (NanoString Technologies, Inc., Seattle, WA, USA), as previously reported (17). This panel assesses 13 cancer-associated canonical pathways related to basic cancer biology (Notch, Wnt, Hedgehog, Chromatin modification, Transcriptional regulation, DNA damage control, TGF-β, MAPK, STAT, PI3K, RAS, Cell cycle, Apoptosis). Briefly, 100 ng of total RNA, quantified by a Qubit Fluorometric System (Thermo Fisher Scientific, USA), from each sample was hybridized for 21 hours at 65°C, followed by purification and RNA/probe complex immobilization in nCounter PrepStation (NanoString Technologies, Inc., Seattle, WA, USA) and cartridge scanning in a digital analyzer (NanoString Technologies, Inc., Seattle, WA, USA), according to the manufacturer’s protocol. Reading with 280 field-of-views (FOVs) was used in the study samples.

### Bioinformatics and Statistical Analysis

We used nSolver™ Analysis Software, version 4.0 (NanoString Technologies, Inc., Seattle, WA, USA) to assess the quality control parameters of all samples. Further analyses were performed using the *R language* and environment for statistical computing (R-project (v3.6.3); The R Foundation, Vienna, Austria) (18). The quantro package (v1.18.0) was applied for cartridge evaluation and to assist in choosing the normalization method. The gene expression data were normalized by the quantile method implemented in the NanoStringNorm package and transformed into a *log2* scale. RNA differential expression was evaluated in the NanoStringNorm package considering two different groups (recurrent *vs.* nonrecurrent low- and intermediate-risk endometrial cancer) with a significance level of *p* ≤ 0.01 and fold change of 2.0 (19). Heatmaps and hierarchical clustering of differentially expressed genes were built with the ComplexHeatmap package (v2.0.0) (20). The STRING database was applied to predict interaction networks from differentially expressed genes (21).

Through the ROC curves, we evaluated the sensitivity and specificity of differential RNA expression by comparing patients with recurrence with those who did not have recurrence using the pROC package (22). An area under the curve (AUC) above 0.7 was considered acceptable for further gene evaluation. We used the backward stepwise logistic regression technique within the MASS package (version 7.3.53) to build a recurrence risk model according to the RNA expression of the samples.

Data analysis was performed using IBM Statistical Package for the Social Sciences (SPSS) database version 27.0 (SPSS, Chicago, IL, USA). Descriptive statistical analysis for quantitative variables used mean, maximum, and minimum and for qualitative variables used percentage. Once the variables were defined, univariate analysis was performed using the chi-square test and Mann-Whitney’s U-test. Variables with a *p* value < 0.2 in univariate analyses were entered into the logistic regression analysis. The threshold for statistical significance was 5%. Study data were collected and managed using REDCap (Research Electronic Data Capture) electronic data capture tools hosted at BCH (23).

## Results

### Patient Features

The clinical and pathological information of the two groups is summarized in **Table 1**. More than 96% of patients are ECOG 0-1. In the recurrence group, we had four patients diagnosed with endometrial adenocarcinoma with squamous differentiation, whereas in the nonrecurrence group, we did not have any diagnosis of this histological subtype (p = 0.01). There was a higher prevalence of white patients in the recurrence group (94.1%) than in the nonrecurrence group (70.6%) (p = 0.075). Other clinical and pathological features were well balanced between the two groups.

**Table 1:** Clinical and pathological characteristics of patients with low- and intermediate-risk endometrial cancer.

		Recurrence (n=17)	Nonrecurrence (n=34)	*p* value
Age (mean)^a^		62.4 (46-77)	62,8 (51-88)	0.779
FIGO staging (%)^b^	IA	11 (64.7)	22 (64.7)	1.00
	IB	6 (35.3)	12 (35.3)	
ECOG Performance Status (%)^b^	0-1	16 (94.1)	33 (97.0)	1.00
	2	1 (5.9)	1 (3.0)	
Ethnicity (%)^b^	White	16 (94.1)	24 (70.6)	**0.075**
	Nonwhite	1 (5.9)	10 (29.4)	
BMI (mean)^a^		31.42 (19.78-43.29)	33.03 (18.67-52.71)	0.873
Smoking history^b^	Yes	3 (17.6)	4 (11.8)	0.673
	No	14 (82.4)	30 (88.2)	
Surgery	With lymphadenectomy	6 (35.3)	16 (47.1)	0.424
	Without lymphadenectomy	11 (64.7)	18 (52.9)	
Surgical route	Laparotomic	8 (47.1)	10 (29.4)	0.233
	Laparoscopic	9 (52.9)	24 (70.6)	
Tumor differentiation grade^b^	Grade 1	9 (52.9)	22 (64.7)	0.417
	Grade 2	8 (47.1)	12 (35.3)	
Histological subtype (%)^b^	Endometrioid	13 (76.5)	34 (100)	**0.010**
	Endometrioid with squamous differentiation	4 (23.5)	0 (0.0)	
Tumor size (mean – cm)^a^		4.5 (2.2-11.5)	3.8 (1.0-7.0)	0.219
Endocervical invasion (%)^b^	Yes	4 (23.5)	7 (20.6)	1.00
	No	13 (76.5)	27 (79.4)	
LVSI (%)^b^	Yes	3 (17.6)	3 (8.8)	0.387
	No	14 (82.4)	31 (91.2)	

BMI, body mass index; ECOG, Eastern Cooperative Oncology Group; FIGO, International Federation of Gynecology and Obstetrics; LVSI, lymphovascular space invasion.

a Mann-Whitney test.

b Fisher’s exact test.

Bold, significant values.

Of the 51 samples that were sequenced for ProMisE, we observed a high frequency of inconclusive results due to the poor quality of the DNA obtained, hampering meaningful analysis. The assessment of MSI was inconclusive in one case, and among the other 50 cases, 12 (24%) were MSI-H. Of the 39 remaining samples for *POLE* sequencing, 18 were inconclusive, and only one (4.8%) was mutated [exon 9 – c.857C>G; p. (Pro286Arg)]. Concerning the 38 samples for *TP53* mutation analysis, 10 cases (66.7%) were wild-type, and five (33.3%) were mutated (**Supplementary Table 1**). There was no difference between the four groups of the ProMisE methodology and the increased chance of recurrence (p = 0.823).

### Gene Expression

**Figure 1:**
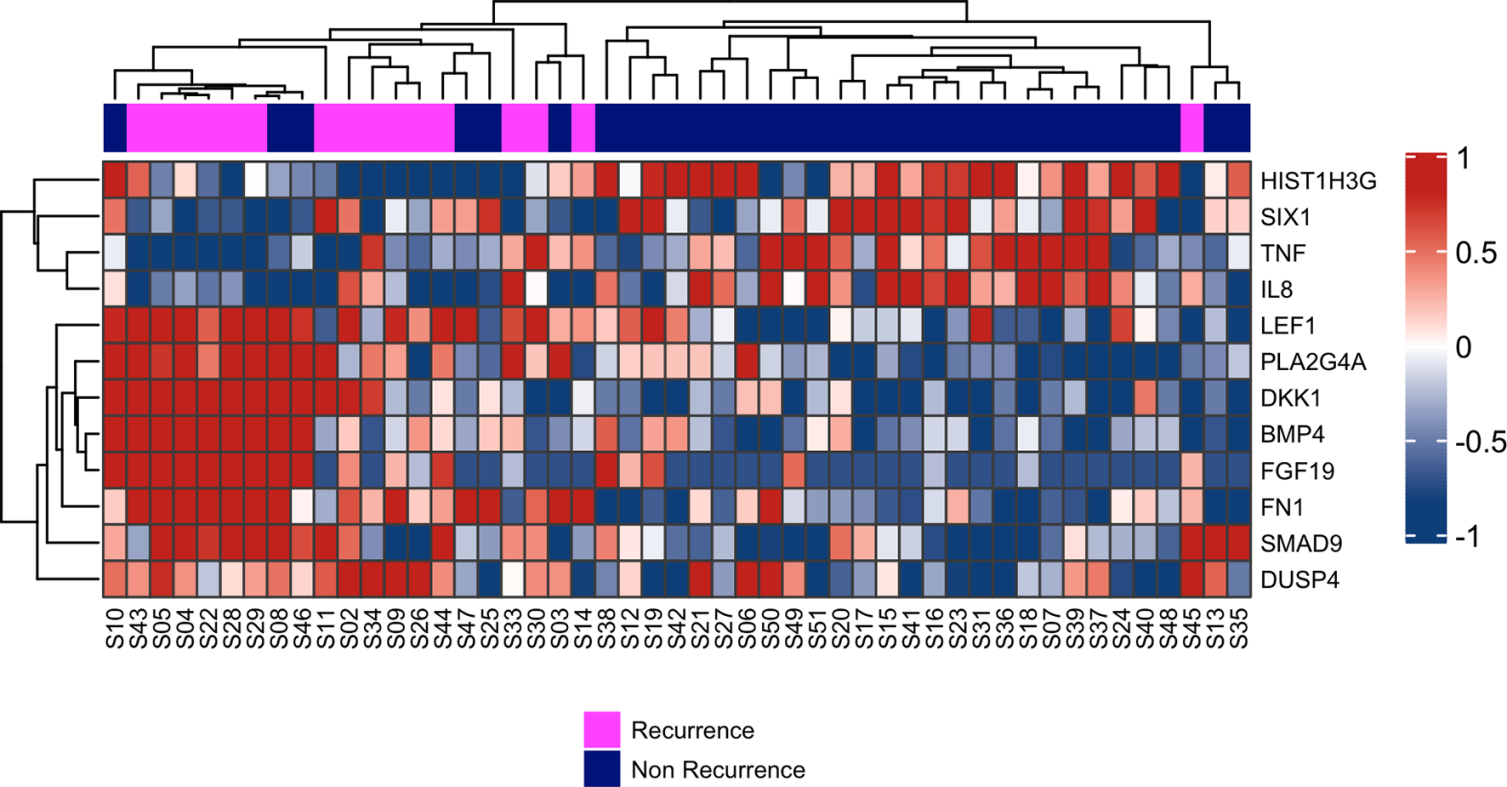
Hierarchical clustering of the 12 RNAs differentially expressed between patients who presented recurrence (pink) compared to those who did not relapse (purple). On the right side: gene expression scaling from dark blue (downregulated) to dark red (upregulated).

Concerning the gene expression profile, 49 of the 51 cases (96%) were conclusive, leading to 16 recurrence and 33 nonrecurrence samples for further analysis. Two samples were excluded due to low-quality RNA. The expression profile based on unsupervised clustering showed 12 genes with differential RNA expression between the two groups studied (**Figure 1**). The *LEF1, PLA2G4A, DKK1, BMP4, FGF19, FN1, SMAD9*, and *DUSP4* genes showed increased RNA expression in the recurrence group, while *HIST1H3G, SIX1, TNF*, and *IL8* were downregulated compared to nonrecurrence group.

We next generated a ROC curve for each of the 12 genes described above to assess the performance of each gene to discriminate between the recurrence and nonrecurrence groups (**Table 2**). All 12 genes presented an AUC higher than 0.7.

**Table 2:** Differentially expressed genes between the recurrence and nonrecurrence groups.

Genes	Fold Change	Sensitivity^1^	Specificity^1^	AUC^2^
***HIST1H3G***	- 2.6	0.7575	0.8125	0.8219
***TNF***	- 2.1	0.7272	0.6875	0.7613
***SIX1***	- 2.1	0.7272	0.75	0.7575
***IL8***	- 2.5	0.6363	0.6875	0.7045
***FN1***	3.0	0.8125	0.8181	0.8532
***DKK1***	5.3	0.6875	0.7575	0.7821
***DUSP4***	2.3	0.75	0.6969	0.7784
***PLA2G4A***	2.3	0.6875	0.8484	0.7575
***LEF1***	2.1	0.8125	0.7272	0.7547
***FGF19***	3.7	0.75	0.7272	0.7537
***SMAD9***	2.1	0.6875	0.8181	0.7348
***BMP4***	3.0	0.75	0.6363	0.7121

^1^Sensitivity and specificity were determined by the Youden index. ^2^AUC, area under the curve.

**Figure 2:**
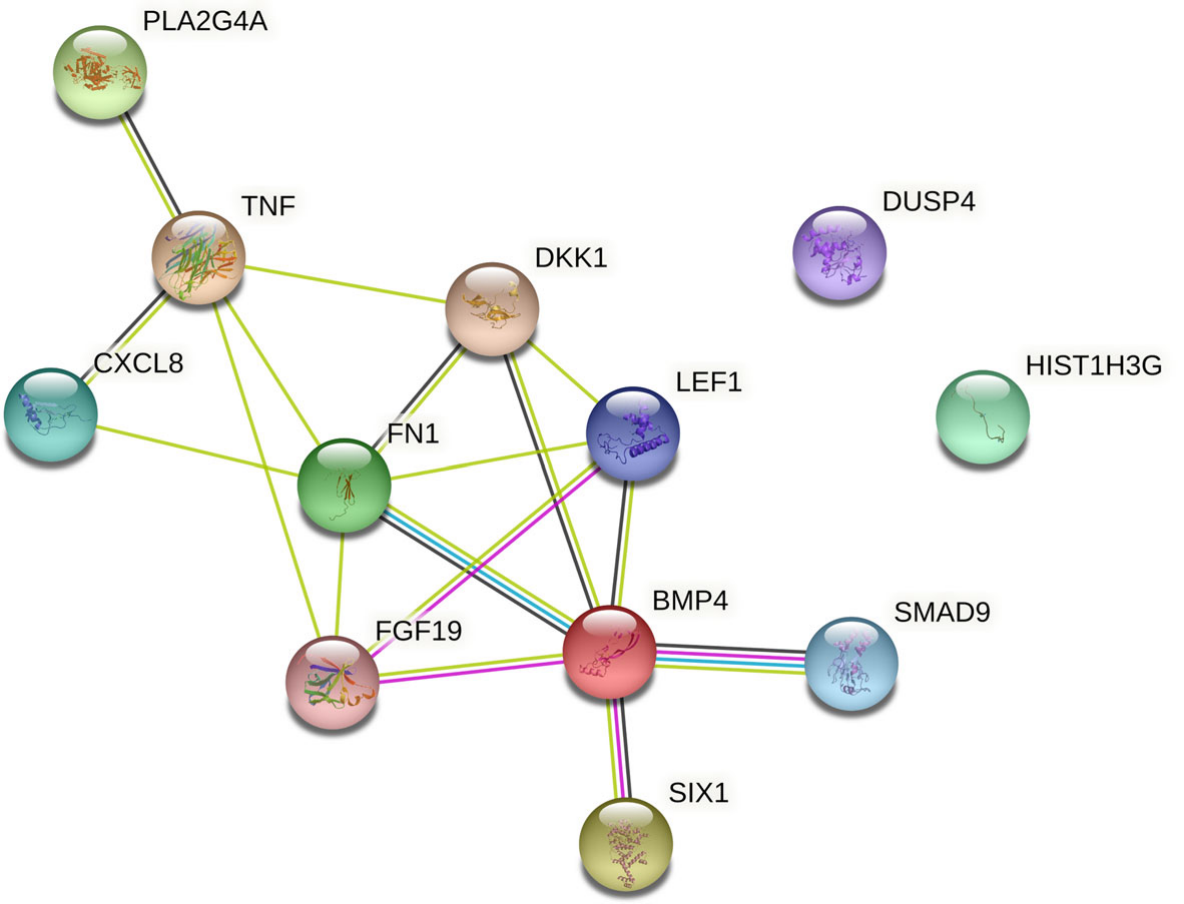
STRING interaction network of the 12 genes differentially expressed in the recurrence and nonrecurrence groups of patients [known interactions (light blue – from curated databases; purple – experimentally determined); predicted interactions (green, gene neighborhood; red, gene fusions; dark blue, gene cooccurrence); others (yellow, text mining; black, coexpression; gray, protein homology)].

To understand the crosstalk among the 12 genes, an interaction network was constructed and is depicted in **Figure 2**. Except for the *DUSP4* and *HIST1H3G* genes, interactions are known among the differentially expressed genes.

### Recurrence Risk Score

Based on the 12 differentially expressed genes, we applied logistic regression to build a recurrence risk score (RRS) and improve predictive performance. Through the backward stepwise logistic regression model, four genes with the best performance were selected: *FN1, DUSP4, LEF1,* and *SMAD9* (increased RNA expression in the recurrence cases). The RRS was calculated as the logit from the logistic regression as follows: RRS = -21.14 + 1.02*FN1 + 1.07*DUSP4 + 0.6211*LEF1 + 0.8832*SMAD9 (**Supplementary Table 2**).

Univariate analysis was performed to calculate the odds ratio (OR) for each gene and for the final score (**Table 3**). Cases with overexpression of the *FN1* gene had an OR of 3.3 for recurrence compared to cases without overexpression. In addition, the final gene score showed an OR of 2.7 for recurrence.

**Table 3:** Selected genes predicting recurrence in low- and intermediate-risk endometrial cancer.

			95% CI		
Genes	Estimates	OR	Lower	Upper	*p* value
***FN1***	1.195	3.303	1.628	6.704	0.001
***DUSP4***	0.960	2.613	1.302	5.241	0.007
***LEF1***	0.818	2.266	1.187	4.325	0.013
***SMAD9***	0.831	2.295	1.223	4.309	0.010
**Score**	1.000	2.718	1.545	4.784	0.001

**Figure 3:**
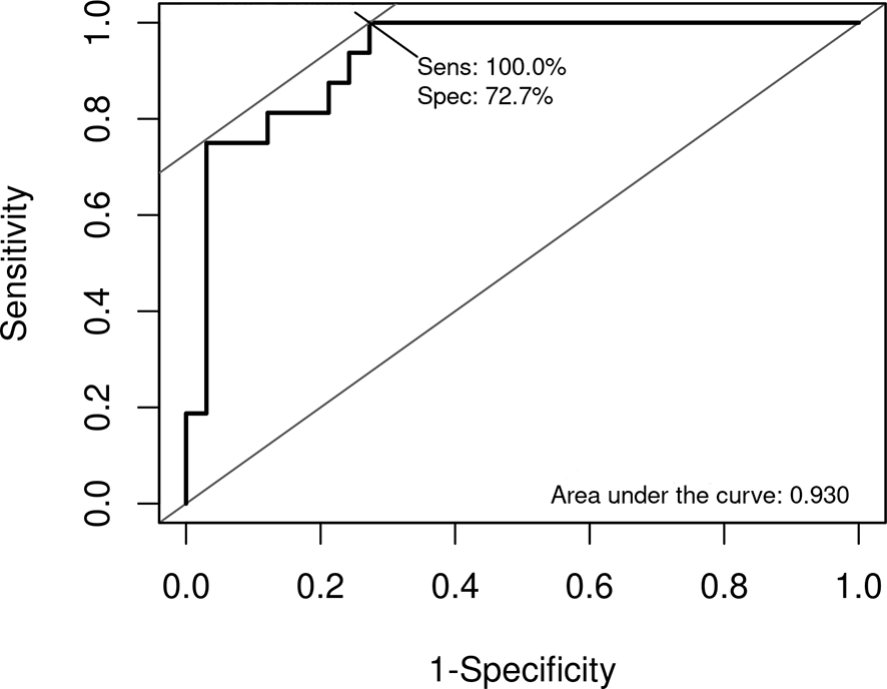
Receiver operating characteristic (ROC) curve of the recurrence risk score (RRS). Sensitivity and specificity were determined by the Youden index.

Moreover, the combination of the expression of the four genes showed an AUC of 0.93, a sensitivity of 100%, and a specificity of 72.7% to identify low- and intermediate-risk endometrial cancer with recurrence trough the RNA expression **(** **Figure 3** **).**

We performed a logistic regression analysis with the four differentially expressed genes score and two significant clinicopathological variables (ethnicity and histological subtype). The histological subtype variable was withdrawn from this model since one of its categories did not contain subjects (no endometrioid with squamous differentiation in the nonrecurrence group), resulting in no data conversion to the odds ratio value. Using a backward stepwise logistic regression technique, a new model was constructed with two parameters: four differentially expressed genes score (OR: 2.616; *p* = 0.001) and white ethnicity (OR: 0.299; *p* = 0.342).

## Discussion

In this study, we characterized the expression profile of two distinct groups (recurrence and nonrecurrence) of low- and intermediate-risk endometrial cancer. Twelve genes were differentially expressed. After performed a logistic regression, four genes remained to define a possible RRS model, exhibiting an impressive AUC of 0.93, with a sensitivity of 100% and a specificity of 73%. To the best of our knowledge, this is the first study to identify a gene signature associated with recurrence in low- and intermediate-risk endometrial cancer.

The four genes are associated with important cancer pathways, namely, the MAPK/PI3K pathways (*FN1* and *DUSP4*), the Wnt pathway (*LEF1*), and the TGF pathway (*SMAD9*).

The Wnt/beta-catenin signaling pathway plays an essential role in tumorigenesis and recurrence in endometrial cancer. Two studies demonstrated the role of the beta-catenin/*CTNNB1* gene as a poor prognostic factor in low-risk endometrial cancer (24, 25). First, in a large study with 342 patients with low-grade and early-stage endometrial cancer through next-generation sequencing, the worst recurrence-free and overall survival was demonstrated in patients with *CTNNB1* and *TP53* mutations (24). In a case-control study similar to ours with recurrent stage I and grade 1 endometrioid endometrial cancers, Moroney et al. showed that *CTNNB1* mutations are present at higher rates in recurrent patients (25).

*LEF1* (lymphoid enhancer factor) is a nuclear transcription factor that interacts with beta-catenin to activate the Wnt pathway (26). The role of LEF1 protein overexpression in the carcinogenesis of endometrial cancer may be related to the modulation of cell surface adhesion proteins, influencing the prognosis of this tumor (27). A study with *LEF1* knockout mice demonstrated its importance in endometrial cancer carcinogenesis. The LEF1 protein is essential in uterine glandular formation, and its overexpression possibly influences the disordered growth of glandular cells and the development of cancer (28).

The MAPK/PI3K pathway is a central pathway in the tumorigenesis of several tumors, and it is even a target in breast cancer treatment (29). The role of *FN1*, which encodes fibronectin, and *DUSP4*, which encodes dual-specificity protein phosphatase 4, in endometrial cancer is not well understood. A recent study by Raglan et al. evaluated the TCGA proteomic data of 560 endometrial cancers and demonstrated that obese patients without cancer had upregulation of several proteins, including DUSP4 (30). Another recent study evaluated the predictive model of lymph node involvement in endometrial cancer using a combined proteomic and transcriptomic approach. The authors reported that high protein expression of fibronectin, cyclin D1, and tumor grade were associated with lymph node involvement. Moreover, overexpression of both *FN1* and *CCND1* (cyclin D1 encoded gene) genes correlated with greater potential for mesenchymal invasion (31).

The third pathway identified was TGF-β through the overexpression of the *SMAD9* gene. This gene belongs to the SMAD superfamily (Drosophila mothers against decapentaplegic protein) made up of important cytokines in the TGF-β family (32). *SMAD9* overexpression is associated with the prevalence of hamartomatous polyposis and is a prognostic factor for lung cancer (33, 34). So far, no studies have addressed its impact in endometrial cancer.

Analyzing the clinical and pathological features of this case-control study, having squamous differentiation in the histopathological diagnosis could be a risk factor for recurrence in this population, according to previously published studies (35, 36). Related to ethnicity, some studies have already shown less medical access in the nonwhite population in the USA impacting oncologic outcomes (37); however, demonstrated a risk due to ethnicity itself. In this study, there was a higher prevalence of white patients in the recurrence group.

Despite these notable findings, our study has some limitations, such as having a small sample size and a retrospective nature. The small number of cases can be explained by the excellent prognosis and low risk of recurrence in low- and intermediate-risk endometrial cancer patients. Therefore, validation of this 4-gene signature in a larger cohort is needed to confirm its predictive value. Moreover, it would be interesting to validate these 4 biomarkers by other methodologies, such as immunohistochemistry. On the other hand, our study has several strengths. First, the robustness of the NanoString methodology proved to be effective for gene expression evaluation in routine samples, even after many years of storage, up to 10 years in our study (38). Second, we evaluated a restricted subpopulation of endometrial cancer to detect risk factors for recurrence in this population. As this is a retrospective study, all patients who relapsed and their matched controls had their pathological reports reviewed by expert gynecologic oncology pathologists to minimize selection bias. Some studies have shown that gynecologic oncology has one of the highest rates of disagreement in the expert pathologist’s report compared to the nonspecialized report (39, 40). In addition, this case-control study represents the experience of a reference cancer center hospital where well-defined treatment protocols minimize possible sample heterogeneity.

## Conclusion

For the first time, we identified a four-gene signature associated with recurrence in low and intermediate endometrial cancer. Additionally, the four genes (*FN1, DUSP4, LEF1,* and *SMAD9*) identified can shed light on the mechanisms of recurrence in endometrial cancer. This study can pave the way for personalized approaches of low- and intermediate-risk endometrial cancer.

## Data Availability Statement

The datasets presented in this study can be found in online repositories. The names of the repository/repositories and accession number(s) can be found below: https://www.ncbi.nlm.nih.gov/geo/, GSE178671.

## Ethics Statement

The studies involving human participants were reviewed and approved by BCH Ethical Review Board in March 2017. Written informed consent for participation was not required for this study in accordance with the national legislation and the institutional requirements.

## Author Contributions

Conception and design: DA, RMR, and RdR. Development of methodology: DA, RMR, and RdR. Acquisition of data: DA. Analysis and interpretation of data: DA, LS, AL, ML, GB, VS, GM, MB, AS, RMR, and RdR. Writing, review, and/or revision of the manuscript: DA, LS, AL, ML, GB, VS, GM, MB, AS, AE, JC, RMR, and RdR. Study supervision: RMR and RdR. All authors contributed to the article and approved the submitted version.

## Conflict of Interest

The authors declare that the research was conducted in the absence of any commercial or financial relationships that could be construed as a potential conflict of interest.

## Publisher’s Note

All claims expressed in this article are solely those of the authors and do not necessarily represent those of their affiliated organizations, or those of the publisher, the editors and the reviewers. Any product that may be evaluated in this article, or claim that may be made by its manufacturer, is not guaranteed or endorsed by the publisher.
