# Supplementary material for: A 4-Gene Signature Associated With Recurrence in Low- and Intermediate-Risk Endometrial Cancer
Source: Front Oncol. 2021 Aug 17;11:729219. doi: 10.3389/fonc.2021.729219 (PMC8416164; doi:10.3389/fonc.2021.729219)
Supplement: Supplementary file 1 [file Table_1.docx]

**Supplementary Table 1.** ProMisE results in recurrence and nonrecurrence groups.

|  | N (%) | Recurrence (%) | Nonrecurrence (%) |
| --- | --- | --- | --- |
| MSI-H | 12/50 (24) | 4/16 (25) | 8/34 (23.5) |
| *POLE* mutated | 1/21 (4.8) | 0/5 (0.0) | 1/16 (6.3) |
| *TP53* wild type | 10/15 (66.7) | 2/4 (50.0) | 8/11 (72.7) |
| *TP53* mutated | 5/15 (33.3) | 2/4 (50.0) | 3/11 (27.3) |

**Supplementary Table 2.** Genes and logistic regression coefficients for the recurrence risk score.

| Genes | Coefficient | Standard error | *p* value |
| --- | --- | --- | --- |
| *Intercept* | -21.1399 | 6.0792 | 0.000506 |
| *FN1* | 1.0188 | 0.4941 | 0.039 |
| *DUSP4* | 1.0663 | 0.4962 | 0.031 |
| *LEF1* | 0.6211 | 0.4399 | 0.157 |
| *SMAD9* | 0.8832 | 0.4531 | 0.051 |
